# Operational Deflection Shapes Magnification and Visualization Using Optical-Flow-Based Image Processing

**DOI:** 10.3390/s21248351

**Published:** 2021-12-14

**Authors:** Adam Machynia, Ziemowit Dworakowski, Kajetan Dziedziech, Paweł Zdziebko, Jarosław Konieczny, Krzysztof Holak

**Affiliations:** 1 Department of Robotics and Mechatronics, AGH University of Science and Technology, Al. A. Mickiewicza 30, 30-059 Krakow, Poland; machynia@agh.edu.pl (A.M.); dziedzie@agh.edu.pl (K.D.); zdziebko@agh.edu.pl (P.Z.); holak@agh.edu.pl (K.H.); 2 Department of Process Control, AGH University of Science and Technology, Al. A. Mickiewicza 30, 30-059 Krakow, Poland; koniejar@agh.edu.pl

**Keywords:** motion magnification, optical flow, image segmentation, modal analysis, cantilever beam

## Abstract

Much information can be derived from operational deflection shapes of vibrating structures and the magnification of their motion. However, the acquisition of deflection shapes usually requires a manual definition of an object’s points of interest, while general motion magnification is computationally inefficient. We propose easy extraction of operational deflection shapes straight from vision data by analyzing and processing optical flow information from the video and then, based on these graphs, morphing source data to magnify the shape of deflection. We introduce several processing routines for automatic masking of the optical flow data and frame-wise information fusion. The method is tested based on data acquired both in numerical simulations and real-life experiments in which cantilever beams were subjected to excitation around their natural frequencies.

## 1. Introduction

The application of computer vision in Structural Health Monitoring (SHM) is an ever-growing field of research. Replacing classical sensors with cameras grants new possibilities and overcomes some disadvantages of conventional methods. Due to the use of computer vision solutions, the limitation of necessary equipment is possible. The set of sensors and network connecting them might be replaced with just one camera. No need for close access, and measuring equipment placement allows saving labor and time. Such a replacement of tools results in an entirely different type of measurement. Instead of analyzing acceleration, displacement is obtained directly by tracking movement on recorded video frames. Usage of a camera allows inspection of hard-to-reach regions remotely, also from a far place using zoom lenses. What is more, it is possible to analyze multiple points simultaneously and select them after recording a video [1]. Relatively easy setup and the large area of inspection are considered to be counterbalanced by a lower precision of measurement comparing to contact non-destructive testing techniques [2]. A crucial part of computer vision applications in SHM is displacement measurement. This area has recently been examined considering abnormal circumstances like intensive sunlight or other unfavorable illumination [3] and different environmental conditions [4].

Recently motion magnification is becoming a powerful method in movement analysis. Its primary purpose is making imperceptible or barely perceptible motion observable. The original technique [5], apart from accurate displacement estimation, bases on the segmentation of pixels into clusters with correlated movement. Then, the selected group’s motion is magnified, and a new video is rendered. Many extensions have been proposed: various signals magnification [6], amplification of tiny motions occurring together with large ones [7] and deep learning application [8]. Especially phase-based approach [9] is important for SHM applications. For image decomposition, complex steerable pyramids are used. Their phase variations are connected with motion, so a new approach for displacement calculation arises. Further, manipulation with these variations may result in movement amplification. This method has been expanded with the use of Riesz pyramids, based on Riesz transform, generalization of Hilbert transform [10]. Despite motion magnification is a fairly new technique, it currently has applications in SHM. Wadhwa et al. [11] presented an example of Mode Shapes (MS) visualization, amplifying vibrations in natural frequencies bands.

Operational Deflection Shapes (ODS) are used to visualize vibration pattern at a given frequency or in a given frequency range for a structure during its operation. Although ODS are not exactly MS, as ODS are a linear combination of MS, they are commonly used when identifying MS are difficult or impossible. ODS have a number of applications in civil engineering [12], industrial rotary machinery [13] and transportation [14]. To obtain an ODS, a simultaneous measurement of vibration responses at a number of locations is required. The higher the number of measurement response locations, the more informative ODS is. During the classical acquisition of vibration data, the number of sensors and data acquisition system input channels has to be equal to the number of measurement response locations, which is a serious limitation for complex structures. To overcome this limitation, the ODS can be collected in a number of measurement runs utilizing one or more reference sensors [13]. This type of measurement can often lead to problems related to the mass loading effect, especially in the case of lightweight structures. Additionally, the collection of vibration responses in a number of runs extends the total measurement time.

Computer vision techniques have been introduced into elements of modal analysis recently. The remote identification of natural frequencies using vision motion analysis with Optical Flow (OF) and image correlation techniques was presented by Caetano et al. [15]. Chen et al. [2] used phase-based video decomposition for displacement obtaining, which grants very high sub-pixel accuracy and further examined frequencies.

MS visualization by color representation, coding phase of movement to hue, and the amplitude to saturation and brightness was introduced by Davis et al. [16]. The authors performed the motion analysis starting from video decomposition using complex steerable pyramids.

The issue was examined further, especially paying attention to small motion. Besides expanded color MS visualization, the authors carried out an estimation of material properties, damping, and extract resonant frequencies [17,18]. Chen et al. [19,20] selected the frame with a maximum deflection after phase-based motion magnification and performed Canny edge detection. Finally, the image was cleaned manually to reduce artifacts and undesirable elements.

Yang et al. [21] after phase-based video decomposition, performed dimension reduction using principal component analysis (PCA). As obtained components still carry information about multiple modes, blind source separation (BSS) was used for their splitting. Only individual modes are magnified, and the images are recomposed. The last step of this method is edge detection. In this technique, preliminary analysis of natural frequencies is not needed.

The method was expanded to allow the analysis of videos recorded at a frame rate not fulfilling the Shannon-Nyquist sampling theorem [22]. Modal identification basing on OF, PCA, and BSS from a video with removed or corrupted frames was proposed by Martinez et al. [23]. An interesting approach for finding natural frequencies is the use of analysis of probability distribution by maximum likelihood estimation [24]. It allows further magnification in a calculated band instead of manually selecting frequencies. Molina-Viedma et al. [25] introduced another representation of ODS. The digital image correlation was used to obtain displacement, which is visualized by the contour plot. The results improve if a video is analyzed after phase-based motion magnification. The method was also extended for 3D applications [26]. The ODS have also been obtained using particle tracking velocimetry, and phase-based motion magnification [27].

Choi et al. [28] proposed the damage detection technique based on the natural frequency comparison. Optimization of an analytical model in terms of damage location and size using a genetic algorithm was performed. The cost function bases on the difference between frequency measured from amplified video and calculated analytically. Phase-based motion magnification was also used for structural monitoring of wind turbine blades [29] and buildings [30]. Comparison of performance of different optical acquisition techniques with accelerometers measurement for experimental modal analysis was performed by Kalybek et al. [31].

Tools proposed in this paper base on OF (original methods: Horn-Schunck [32] and Lucas-Kanade [33,34]), which is fundamental motion analysis technique in computer vision. OF describes the movement of pixels between consecutive frames of video. This method is based on pixels’ brightness constancy assumption. Advanced displacement measurement using Deepflow has been proposed by Won et al. [35]. Javh et al. [36] used OF for MS recognition analyzing the amplitude of estimated displacements. Color mapping of motion was performed by Kah and Narroschke [37] for visualization purposes allowing simple results assessment. HSV color space (consisting of three channels denoted as H for hue, S for saturation, and, finally, V for value) conversion of OF results for further processing has been presented by Du et al. [38]. The authors used transformation into HSV and then into RGB color space to perform object tracking.

In addition to the methods presented above, we present a set of novel OF processing tools allowing straightforward displacement visualization, motion masking, and ODS magnification. We obtain a binary mask of moving regions, analyzing the periodicity of movement and performing k-means segmentation. This region mapping can be used for area selection for motion magnification. Presented displacement visualizations are based on converting OF using HSV and L*a*b* color spaces (L* stands for luminance, a* describes shades between green and red, and b* denotes tones of blue and yellow). Color representation grants a clear, uncomplicated movement description. Unlike previous approaches [16,17,18] our relies on a straightforward OF transformation and is used for further preparation of advanced depiction. Finally, we demonstrate one-dimensional ODS visualization, which is an alternative novel motion magnification method.

In Section 2 detailed description of proposed techniques is provided. Next, in Section 3 results of the application of developed processing tools are presented. The simulation and the experimental test case are described. Finally, Section 4 is conclusions.

## 2. Materials and Methods

Developed tools allow for straightforward motion analysis, giving new visualization capabilities, basing on OF computation. They consist of three components enabling binary masking of moving regions, color visualization of motion, and magnifying ODS. In this section, each of them is described in detail. A simulation of a cantilever beam setup excited in its third natural frequency (208 Hz) will be used to visualize the algorithms’ principles of operation. The details of the simulation setup will be provided in Section 3.1. All processing was performed using software prepared in Matlab. The source code is available on demand from the corresponding author.

### 2.1. Binary Mask and Movement Direction

The first processing step is the preparation of a binary mask of moving regions. Its purpose is to mark an area with objects in motion to use this information for further analysis. Such region indication allows clarity enhancement of results in subsequent stages of processing. Furthermore, in this step, the main direction of movement is found, which is used in preparing visualizations. Simple OF magnitude thresholding have limitations such as promoting the largest shifts and the inability to separate different moving objects. Therefore binary mask is prepared based on OF direction changes counting. Input data for this method is a raw video presenting an oscillating object. This video processing aims to indicate moving objects or their parts, during related or similar movements, especially with the same frequency. Results of OF direction changes might be further processed using normalization and thresholding or by segmentation.

#### 2.1.1. Main Direction of Movement

**Figure 1:**
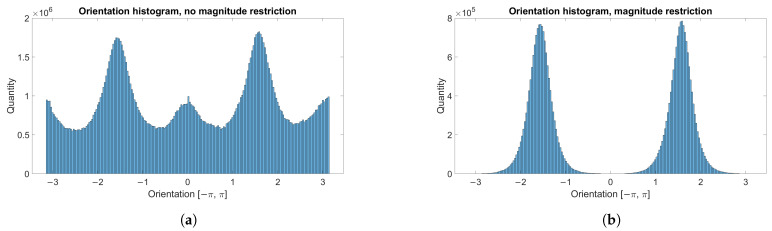
Orientation histogram: (**a**) without magnitude restriction, (**b**) magnitude restricted to greater than 0.1.

**Figure 2:**
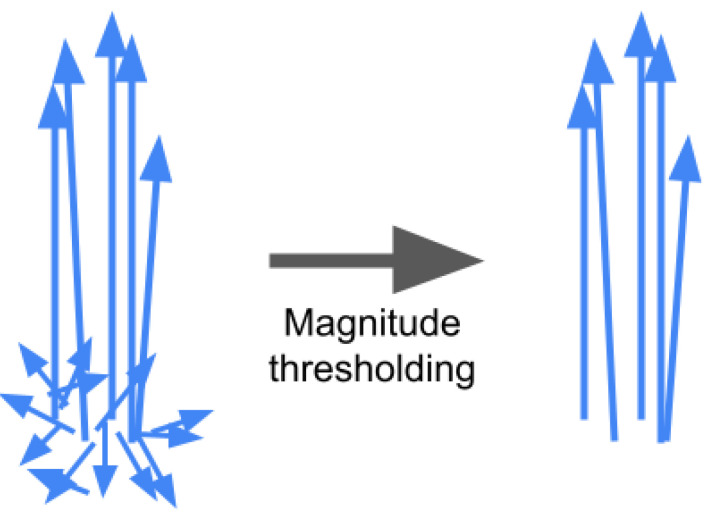
Irrelevant, noisy motion vectors tend to be shorter, and omitting them by magnitude thresholding helps to distinguish the dominant direction of movement.

Determining the main direction of movement is crucial for obtaining further visualizations. OF is calculated for each point of the frames in the whole video and stored. OF is computed from grayscale images, although it might be obtained from images in the RGB color space. Then OF orientation from all frames and all points is sorted into bins of size $\frac{\pi }{100}$. An exemplary histogram for the movement of a simulated beam is shown in Figure 1a. Irrelevant, noisy motion vectors tend to have a smaller magnitude in comparison to the vectors characterizing the main motion. As the intention is to determine the dominant orientation of movement, an additional restriction is applied. If OF magnitude is less than 0.1 orientation is not taken into account. Schemetically this process is shown in Figure 2. Figure 1b shows histogram including this constraint. This limiting threshold and size of bins have been set experimentally. Finally, the midpoint of the bin with the largest number is chosen as the main direction of movement in the video.

#### 2.1.2. OF Direction Changes

Having OF calculated, results obtained for two consecutive frames of a video are compared to detect movement direction changes. For each pixel, the number of OF sign changes with respect to the vertical axis is counted. Method’s accuracy has been increased using two additional constraints that have to be fulfilled at a particular time for a specific point to be analyzed.

- OF magnitude must be greater or equal to 0.01.
- OF orientation is restricted to the regions of the previously determined main direction and to the opposite direction. Angles difference has to be smaller than $\frac{\pi }{4}$.

Values of these thresholds have been set experimentally based on real case data (see Section 3.2). These limitations help to distinguish the main object of interest.

The simplest way of obtaining a binary mask is normalization by dividing all values by their maximum and thresholding. This approach has some drawbacks. The first is the possible presence of fragments of undesirable elements that happen to move in the main direction by coincidence. The second one is substantial discontinuities in the binary mask, which might be caused by occlusions, vibration nodes, or imperfections in the acquired data.

#### 2.1.3. Segmentation and Masks Combination

Segmentation is used to obtain a binary mask instead of normalization and thresholding. In this case, clusterization with the k-means method is applied. Only values representing the sum of OF direction changes are analyzed regardless of position in the image. The number of clusters is set to three for separating the object of interest, other undesirable elements, and background.

**Figure 3:**
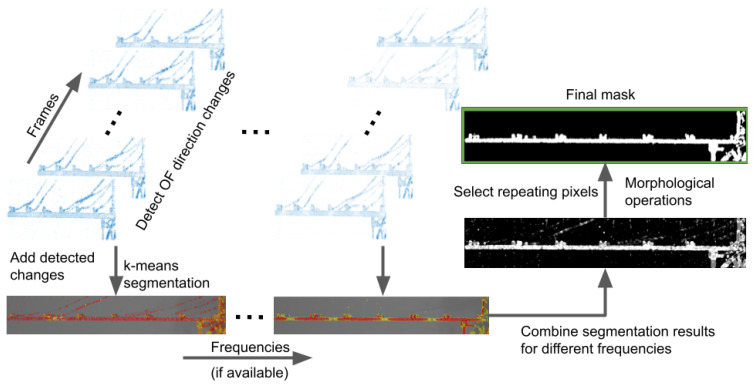
Scheme of mask preparation. OF direction changes among frames are detected and segmented. Data are combined for different frequencies, repeating pixels are selected, and morphological operations are performed.

If an analyzed object is recorded multiple times while moving with different frequencies, the beneficial approach is combining masks obtained in different conditions. Especially it helps to handle discontinuities in nodes of vibration at various natural frequencies. Multiple case analysis allows the preparation of the final mask by choosing pixels present on a certain number of individual instances. For example, the point of an image must be present on at least half of the masks to be placed on the resulting one. Further, in more difficult cases, morphological operations might be useful to increase accuracy. Figure 3 shows the whole process of obtaining a binary mask using real case data (see Section 3.2).

### 2.2. Color Visualization

This stage can be conducted independently or use previously obtained binary mask and main direction of movement. Processing starts with OF. To prepare color visualization, the following steps are taken. OF orientation and magnitude for each point in the image are transformed to obtain a new representation. The orientation is rotated, so the main direction of movement (determined in the previous step or set manually) will be represented by the value of $-\frac{2\pi }{3}$. It is essential for the preparation of ODS visualization in the next stage and will be justified in Section 2.3. The original orientation range is [−π,π], thus values are shifted by adding π and then divided by 2π, so the final range is [0,1] and relations between values are retained. Transformed orientation is considered as a hue in HSV color space.

OF magnitude is scaled into range [0,1]. The new value $m_{r}$ is obtained by subtracting minimum from the old value *m* and dividing by difference between maximum and minimum:

(1) $$m_{r}=\frac{m-min}{max-min}.$$

**Figure 4:**
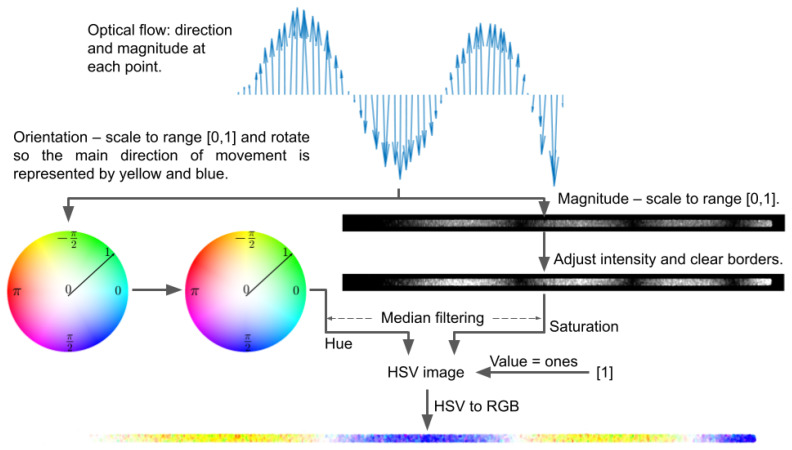
Scheme of color visualization. OF orientation is mapped as hue and magnitude as saturation in HSV domain in order to obtain color representation of movement.

The minimum and maximum are the smallest and the largest magnitude in all image points at a certain time. Further, magnitude treated as a grayscale image is adjusted by saturating the bottom and the top 1% of values and mapping the other ones to the full range to increase contrast, making final visualization more transparent. The transformation is appropriate considering that relative displacements are of interest. Moreover, a three-element wide border is set to zero to reduce noise at the edges of the image. Converted values are interpreted as saturation in the HSV domain. The further noise reduction is conducted by applying the median filtering of hue and saturation with a window of size $W_{1}$, in this research set to 3×3. The third component–value is completed with ones. OF orientation and magnitude are represented by the color of each point. In order to show or save the HSV-based result, the RGB color space is used. The entire conversion process is schematically shown in Figure 4.

### 2.3. ODS Visualization

The most important part of the described processing is ODS visualization based on OF transformation with HSV color space usage. To increase the capabilities of the method, a previously obtained binary mask is applied. The area outside indicated region becomes the white background. This step allows the extraction of an object of interest and reduces the impact of noise.

This stage of processing includes the next color transformation and starts with the conversion of previously obtained representation (Section 2.2) from RGB to L*a*b* color space. Further, only the b* component is analyzed, thanks to the coding of the main direction of movement with yellow and its opposition with blue. Before further processing, rotation of the image is necessary in order to place the yellow-blue direction vertically. Component b* range is [−128,127], with negative values representing blue and positive yellow. From each column of this channel, the mean is calculated. Averaging is performed only from nonzero elements. Therefore, the background has no impact on estimation. The resulting vector describes the ODS. Further, the moving average is calculated over it, using a window of size $W_{2}$. In this research, based on preliminary tests, $W_{2}$ is set to 150. To increase accuracy, the moving mean can be calculated over a vector limited to the object’s length, reducing the background’s impact. For this purpose, the shape can be restricted according to the previously obtained binary mask or by neglecting part on the side of the image, where all values of the b* component are equal to zero. For visualization purposes, the result might be multiplied by a number set by a user, which allows increasing or reduction of a shift in the next step. The shape is rounded to integers. Obtained vector’s length is equal to the number of columns in the original image. To build a new representation of ODS, points indicated by the binary mask are shifted by the values in the calculated vector. The empty space after the moved points is filled in with a white background.

**Figure 5:**
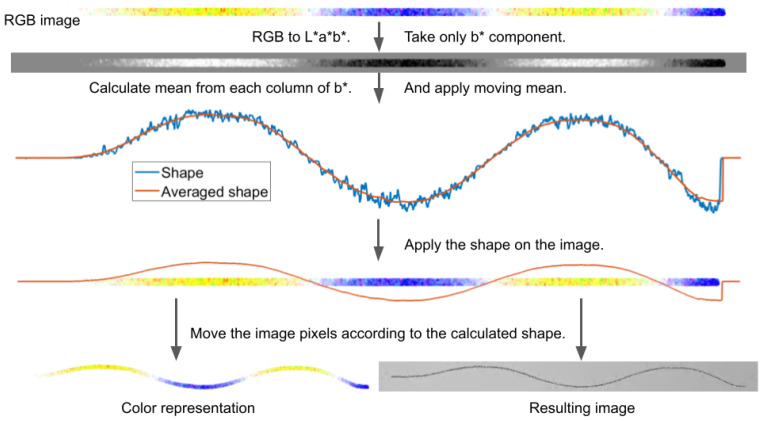
Scheme of ODS visualization. Shape obtained from b* component of L*a*b* domain allows determining pixels shift to visualize mods.

Additionally, ODS magnification on raw video frames is performed. For this purpose, pixels indicated by the binary mask are shifted in accordance with the transformation of color visualization. Resultant blank spaces have to be refilled. In these regions, inward image interpolation is performed. Figure 5 illustrates the entire procedure of ODS visualization.

## 3. Results and Discussion

In this part, the results of the application of described techniques are presented. The first case demonstrates the simulation of movement of a cantilever beam and the second example is the real test case. All results have been obtained using Farnebäck’s method [39] for OF computation.

### 3.1. Simulation

**Figure 6:**
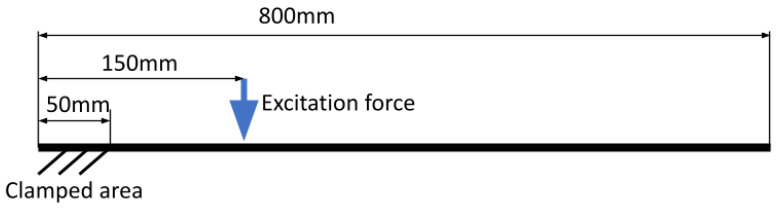
Scheme of the simulated cantilever beam.

In this example movement of the cantilever beam is simulated. The object is 800 mm long, 40 mm wide, and 4 mm thick. The beam is fixed at the length of 50 mm (Figure 6). The model was prepared in Altair HyperMesh using hexahedral FEM mesh of size 1×1×4 mm. First, the computational modal analysis was computed to identify the natural frequencies of the structure. Next, the Dynamic Transient Modal Superposition simulation was conducted. This was done independently for each particular mode shape considered in the individual simulation. In each case, the impulse force of 100 N lasting 0.002 s excited the structure. Thanks to the Modal Superposition approach only the required mode shape was taken into account in the transient response, not introducing other modes. Next, the FEM simulation results were used to create animation using the Blender graphics program. This procedure is discussed by the authors in their recent paper [40].

**Figure 7:**
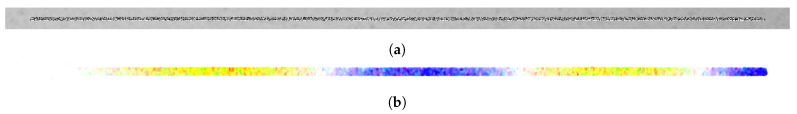
Simulation: (**a**) raw frame, (**b**) color representation for 208 Hz.

Figure 7a presents raw frame and Figure 7b shows color visualization of movement. The main direction of movement is vertical. Yellow regions indicate parts moving upwards at this moment and blue–downwards.

**Figure 8:**
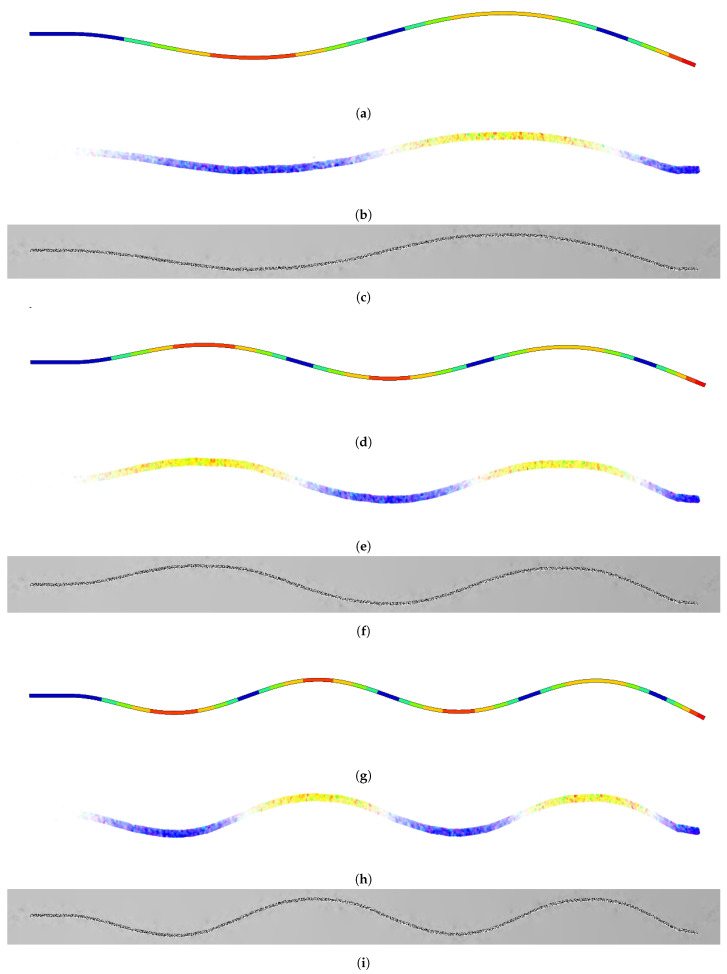
ODS visualization: FEM simulation results (colors are unrelated to the proposed visualization), color representation and original frames transformation, according to the transformation procedure presented in Figure 5: (**a**–**c**) 106 Hz, (**d**–**f**) 208 Hz, (**g**–**i**) 345 Hz.

As the video presents only the beam and background, the use of the binary mask is unnecessary. Also, the direction of movement is vertical, so any rotation is not required. Figure 8 shows ODS visualization for different frequency cases: 106 Hz, 208 Hz, 345 Hz. Color representation and raw frame transformation are set together with FEM simulation results.

### 3.2. Experimental Case

**Figure 9:**
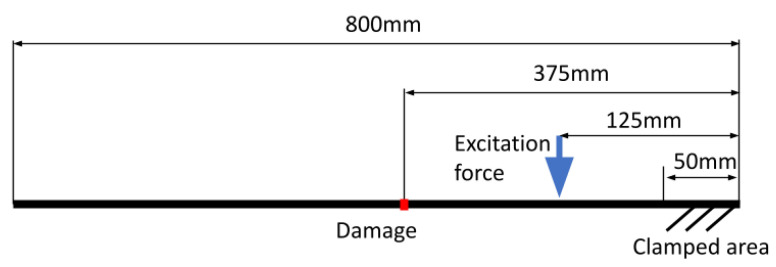
Scheme of the examined cantilever beam.

In this example, recording of the aluminum cantilever beam with dimensions of 800×40×5 mm, fixed at one end, is analyzed. The beam is damaged by a 20 mm notch. Figure 9 shows the beam schematically. Five tests have been conducted. The first test was with the use of white noise excitation to identify the natural frequencies. The remaining tests were conducted with the use of a sine excitation at the beam’s resonant frequencies. Video is obtained with the use of a high-speed camera Phantom V9 with Carl Zeiss lens (focal length 50 mm, f-number f/5.6) at 1000 frames per second. Face side of the beam was covered in a random speckle pattern to allow OF calculation.

**Figure 10:**
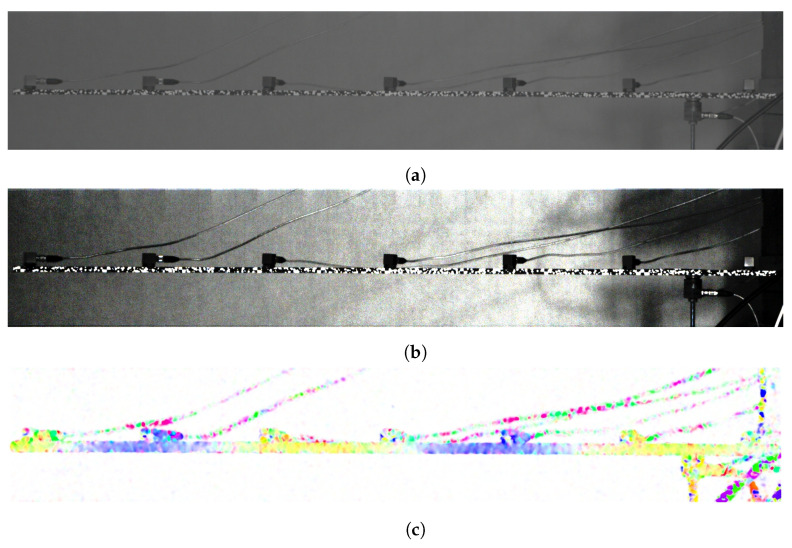
Real object example. (**a**) Raw image of the beam. (**b**) Image after histogram equalization–only for visualization purposes, not used during processing. (**c**) Color visualization of the beam movement: yellow parts are moving up and blue down.

The original frame is presented in Figure 10a. As this image is not very clear, for visualization purposes, histogram equalization has been performed—Figure 10b. Such processing has also been applied to present further results. Color representation for sine 347 Hz excitation is shown in Figure 10c. This visualization allows the user to distinguish moving regions and describes the direction and relative magnitude of imperceptible movement. In this case, as the direction of the beam movement is vertical, blue regions indicate parts moving downward and yellow upward.

**Figure 11:**
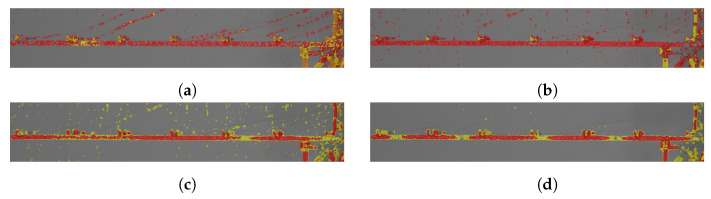
Results of k-means segmentation. Gray area is the background. To cover the object accurately different number of clusters were chosen depending on case. (**a**) 40 Hz–four clusters, three selected (red, orange, yellow) (**b**) 110 Hz–three clusters, one selected (red) (**c**) 209 Hz–three clusters, two selected (red, yellow) (**d**) 347 Hz–three clusters, two selected (red, yellow).

The process of obtaining a binary mask starts with OF direction changes counting and k-means clustering of its results. Segmentation outcomes overlaid on original frames are presented for different frequencies in Figure 11. The number of clusters is set to three, except in the case of 40 Hz excitation (Figure 11a), where it is set to four and three of them are selected. A relatively low frequency of movement causes this situation. In the 110 Hz case (Figure 11b), one of three clusters is chosen and in 209 Hz (Figure 11c) and 347 Hz (Figure 11d) examples, two are selected. This approach is used in order to cover a larger area. Segmentation results vary depending on the frequency of analyzed movement. In the case of 40 Hz excitation, numerous elements, e.g., elements of wires, are present in a cluster representing the main parts of the beam. As frequency increases, these elements are neglected. Finally, the case of 347 Hz sine shows significant difficulty, which is possible omitting regions neighboring nodes. In these areas, movement magnitude is low, resulting in the classification of these parts as other objects or even as background closer to nodes.

**Figure 12:**
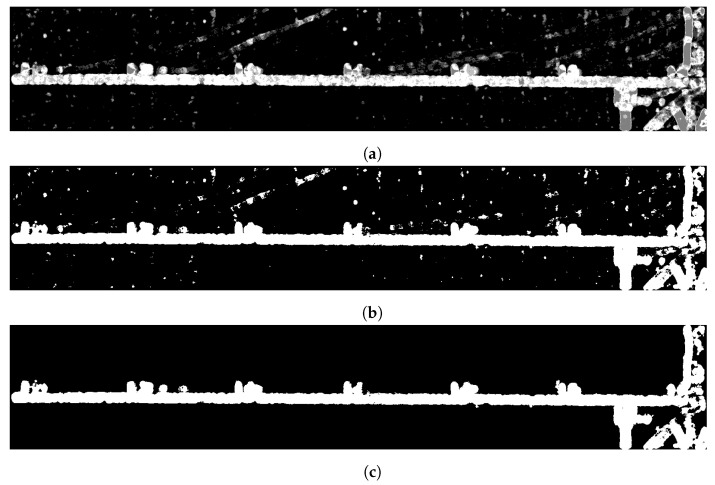
Binary mask preparation: (**a**) sum of binary masks for different cases (pointed in Figure 11), (**b**) selection of points present at at least two masks, (**c**) final mask–only main object selected.

Further, results obtained for different frequencies are combined. Their normalized sum is shown in Figure 12a. Next, only points present in at least two cases are retained (Figure 12b). Again, the threshold is relatively low, so a greater region is included. Finally, only pixels connected into one largest object are preserved (Figure 12c). Despite the shortcomings of singular binary masks, their combination grants sufficient object indication.

**Figure 13:**
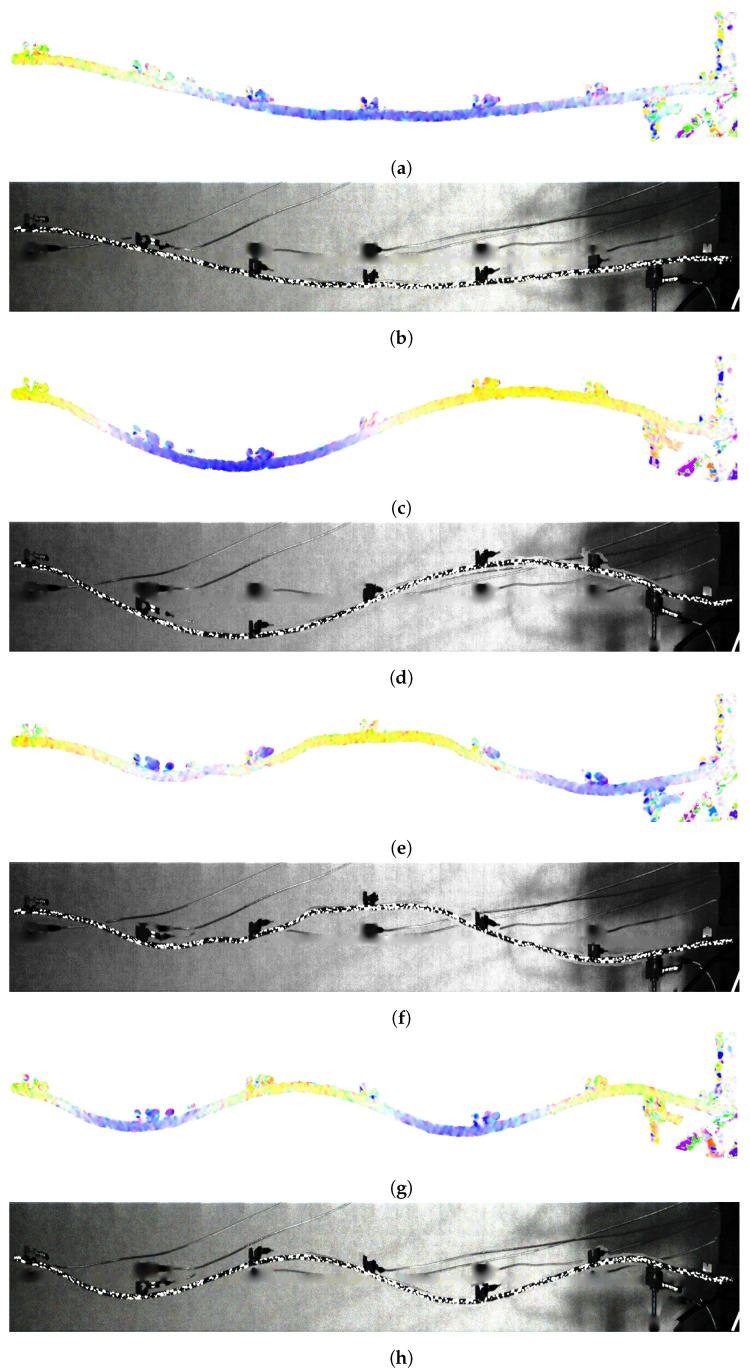
ODS visualization: color representation and original frames transformation after histogram equalization: (**a**,**b**) sine 40 Hz, (**c**,**d**) sine 110 Hz, (**e**,**f**) sine 209 Hz, (**g**,**h**) sine 347 Hz. The remaining parts of accelerometers were placed outside the binary mask.

Figure 13 shows ODS visualization for the aforementioned four different frequencies. Color representation and raw frames transformation, both obtained without additional multipliers, are set together. Results have been obtained using the binary mask presented in the previous step. Color visualization is suitable and clear for the user. On the other hand, raw transformation shows the real scene, which is also beneficial. An important inconvenience is the presence of additional objects, e.g., accelerometers, reflecting on the obtained visualization. In this case, sensors have become an integral part of the object. However, their influence is noticeable and reduces the smoothness of ODS.

## 4. Conclusions

We have presented a set of tools allowing motion analysis using high-speed camera recordings. Preparation of binary mask based on movement periodicity helps to indicate objects or their parts vibrating similarly and reject irrelevant elements. Color transformation of OF results in a simple representation of movement, especially including very small motion. The main achievement is novel ODS magnification based on the image transformation. Proposed tools have been assessed on the simulation data and real case recording presenting a vibrating cantilever beam. The principal advantages of described techniques are their simplicity, clarity, and no need of performing motion magnification for movement analysis. As a serious drawback might be pointed one-dimensional magnification of ODS.

## Data Availability

Data used in this article are available on-demand from the corresponding author.

## Abbreviations

The following abbreviations are used in this manuscript:

SHM	Structural Health Monitoring
MS	Mode Shapes
ODS	Operational Deflection Shapes
OF	Optical Flow
PCA	Principal Component Analysis
BSS	Blind Source Separation
